# Advances in the Chemical Analysis and Biological Activities of Chuanxiong

**DOI:** 10.3390/molecules170910614

**Published:** 2012-09-06

**Authors:** Weixia Li, Yuping Tang, Yanyan Chen, Jin-Ao Duan

**Affiliations:** Jiangsu Key Laboratory for High Technology Research of TCM Formulae, Nanjing University of Chinese Medicine, Nanjing 210046, Jiangsu, China

**Keywords:** Chuanxiong, organic acids, phthalides, alkaloids, polysaccharides, ceramides, cerebrosides

## Abstract

Chuanxiong Rhizoma (Chuan-Xiong, CX), the dried rhizome of *Ligusticum chuanxiong* Hort. (Umbelliferae), is one of the most popular plant medicines in the World. Modern research indicates that organic acids, phthalides, alkaloids, polysaccharides, ceramides and cerebrosides are main components responsible for the bioactivities and properties of CX. Because of its complex constituents, multidisciplinary techniques are needed to validate the analytical methods that support CX’s use worldwide. In the past two decades, rapid development of technology has advanced many aspects of CX research. The aim of this review is to illustrate the recent advances in the chemical analysis and biological activities of CX, and to highlight new applications and challenges. Emphasis is placed on recent trends and emerging techniques.

## 1. Introduction

Chuanxiong Rhizoma (named as Rhizoma Chuanxiong before 2010 in the Chinese Pharmacopoeia), the dried rhizome of *Ligusticum chuanxiong* Hort., known as Chuan-Xiong (CX) in folk medicine, and belonging to the Umbelliferae family, is one of the oldest and most popular herbal medicines in the World [1,2]. It has been used for thousands of years in traditional Chinese, Japanese, and Korean folk medicine [3]. It was firstly cited as “Xiong-Qiong” in Shennong’s *Classic of Materia Medica* (200–300 A.D., Han Dynasty) an oldest Chinese herbal classical masterpiece [1,4]. Later, it was cited by the name “Chuan-Xiong”, which has been used until today, in *Yixue Qiyuan* (Jin Dynasty, Zhang Yuansu) [5,6]. This herb is otherwise known as Senkyu (Jananese), Ch’onkung (Korean), and Szechuan lovage root (English), respectively. Senkyu, the dried rhizome of *Cnidium officinale* Makino (syn. *L. officinale* Kitagawa, Umbelliferae) which is the original plant of the important crude drug CX in China, is one of the most frequently occurring drugs in the prescriptions of traditional Chinese medicines (TCMs) used in Japan [7,8,9,10]. According to study, *C. officinale* and *L. chuanxiong* are closely related species with 98% sequence identity [11]. Some studies have shown that both of them had antioxidative activities, through their high free radical scavenging ability, they may exert vascular relaxant effect, and inhibitory effects on DNA damage and apoptosis induced by ultraviolet B in mammalian cells [12,13,14]. However, it is *L. chuanxiong* that is recorded by the Chinese Pharmacopoeia and applied commonly in China.

CX is an annual herb, and its growing period can be divided into six stages (Table 1). From ancient times until today, Chuanxiong Rhizoma has been harvested each year in late May. Furthermore, studies [15,16,17] also showed that the optimal harvest time for this herb is in the period from the middle of April to the end of May. The resources of CX are very rich and widespread, with the main production areas being Dujiangyan (original name: Guan County), Pengzhou, Xindu, and Chongzhou, Sichuan province, China. CX cultivated in Dujiangyan is traditionally recognized as the authentic and superior herb [18,19,20].

**Table 1:** The growth and development stages of CX.

Stage	Aug.	Sept.	Oct.	Nov.	Dec.	Jan.	Feb.		March		April		May
Seeding													
Stem emergence and growth													
Senescene													
Emergence of the secondary stems													
Tillering													
Rhizome expansion													

CX is warm in property and pungent in flavor, with the functions of activating qi, promoting blood circulation, expelling wind and alleviating pain, which has high medicinal value [21]. It is one of the major clinically used cardiovascular protective TCMs and a popular medicine widely used in prescriptions for the treatment of atherosclerosis [22], vasodilatation [23], thrombus formation [24], ischemic stroke [25,26], angina pectoris [27], and hypertension [28,29], because of its reputation for thousands of years in China of facilitating blood circulation and dispersing blood stasis [30,31]. Additionally, it also has antioxidant [7,14], neuroprotective [32,33], antiinflammatory [34], antibacterial [35], antiproliferative [36], and proapoptotic activities [37]. What is more, its medicinal value has been demonstrated by numerous experiments, pre-clinical studies and clinical trials [33,35]. Furthermore, it is widely applied in food preparation as a health protection, usually added to a soup, such as CX mutton soup and CX fish head soup [38]. It is not only used for medicinal purpose, but also for health care products, facial cosmetics, as a forage additive, tobacco flavor additive, natural preservative, and so on [39].

These bioactivities are attributed to the chemical constituents of CX. So far, more than 200 compounds have been isolated from this herb, more than 80 compounds of which belong to various different structural types that have been identified. These compounds can be grouped into five basic types believed to be responsible for the bioactivities of CX, namely phenols and organic acids, phthalides, alkaloids, polysaccharides, ceramides and cerebrosides. Except for these five major types, CX also includes mineral elements, and other types of compounds. The contents of these constituents may vary significantly due to geographic sources, harvesting and processing, and thus affect the therapeutic effects of CX. Therefore, comprehensive quality control is critical to ensure the efficacy and safety in clinical use. Due to the complicated chemical composition, sensitive, accurate and high-resolution analytical methods should be established for the simultaneous qualitative and quantitative analysis of constituents from CX. What is more, chemical analysis of TCMs is an important subject in biochemical, pharmaceutical and clinical researches.

In the past two decades, rapid development of technology has advanced many aspects of CX research. Therefore, in this review, we summarize the progress in chemical analysis and pharmacology of the major five types of natural products from CX, and mainly focusing on the chemical structures, isolation, qualification, quantification, and biological activities. Due to the importance of fingerprint analysis [40,41], and the rich and wide spread of CX, the development of the chromatographic fingerprint of this herb is also discussed.

## 2. Chemical Compounds and Bioactivities

### 2.1. Phenols and Organic Acids

#### 2.1.1. Chemical Structures

**Figure 1:**
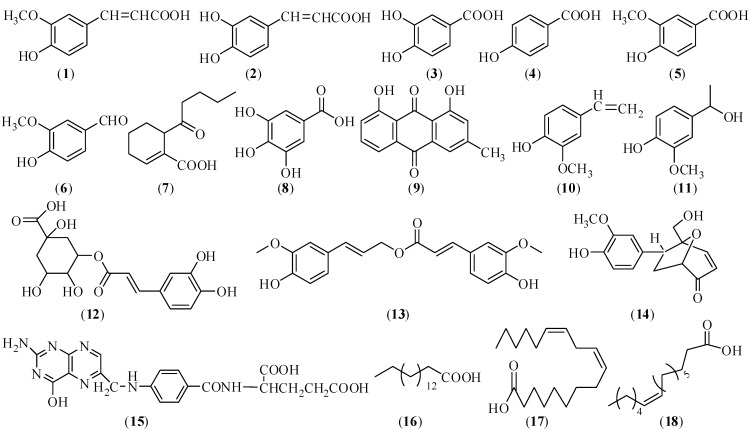
Chemical structures of the identified phenols and organic acids in CX: (**1**) ferulic acid; (**2**) caffeic acid; (**3**) protocatechuic acid; (**4**) p-hydroxybenzoic acid; (**5**) vanillic acid; (**6**) vanillin; (**7**) sedanonic acid; (**8**) gallic acid; (**9**) chrysophanol; (**10**) 3-methoxy-4-hydroxystyrene; (**11**) 1-hydroxy-1-(3-methoxy-4-hydroxyphenyl)-ethane; (**12**) chlorogenic acid; (**13**) coniferyl ferulate; (**14**) 5-hydroxymethyl-6-*endo*-3-methoxy-4-hydroxyphenyl-8-oxa-bicyclo(3.2.1)-oct-3-one; (**15**) folic acid; (**16**) palmitinic acid; (**17**) linoleic acid; (**18**) sinapic acid.

Phenols and organic acids are one kind of the major characteristic constituents in CX. So far, at least 18 phenols and organic acids have been obtained from this herb. The main chemical structures of these phenols and organic acids (compounds **1**–**18**) are given in Figure 1 [42,43,44,45,46,47,48,49].

#### 2.1.2. Sample Preparation for Chemical Analysis

CX contains different types of compounds, among which there are hydrophilic or hydrophobic, polar or nonpolar ones. Therefore, good sample preparation methods are necessary to ensure that most compounds are efficiently extracted. Several methods have been reported for the separation of some organic acids from CX, including thin layer chromatography (TLC) [50], reversed-phase high performance liquid chromatography (RP-HPLC) [51,52], HPLC-mass spectrometry (HPLC-MS) [53], capillary zone electrophoresis (CZE) [54], and immobilized liposome chromatography for compound **1** [55], HPLC-MS for compound **13** [56], gas chromatography-MS (GC-MS) for compounds **1** and **6** [57], and capillary electrophoresis (CE) for compounds **1**, **2** and **12** [58]. Zhao *et al*. [59] developed a RP-HPLC method for the determination of five kinds of phenolic acids in CX: **1**, **2**, **3**, **8**, and **12**. And on the basis of this method, compounds **1** and **2** are found to be the main phenolic acids in CX. Up to now, most literatures about organic acids from CX focus on the analysis of compound **1**. More important, compound **1** is found to exist in free form, and **2** in esterified or insoluble-bound form. The phenolic acid, particularly compound **8**, is unstable under alkaline conditions in air, and it is necessary to hydrolyze the sample under argon or nitrogen [60]. However, publications about other acids in CX are very few. Otherwise, several analysis methods for phenolic acids have been set up for a variety of other samples, such as Danggui [61], fruit [60], rice [62] and wine [63]. These works successfully demonstrate that, by optimizing the parameters for the eluent or carrier system, the high-resolution separation of a complicated acid mixture can be easily achieved.

#### 2.1.3. Quantitative Analysis

Compound **1**, one of the main effective components in CX, is widely used as one of the marker compounds to assess the quality of CX and its products [64,65]. In the reported literatures, the concentration of **1** in CX varies within the range of 0.107–2.374 mg/g, quantified by a variety of methods (shown in Table 2) [42,51,66,67,68,69,70,71,72,73,74,75,76]. Additionally, it was also determined by other methods such as near-infrared spectroscopy (NIRS) [77]. Apart from the variation in natural abundance among the herb samples, the nature of extraction solvents and methods are likely to be a critical cause. CX sample is commonly extracted using a variety of solvents, namely: ethanol, methanol, methanol-water-36% acetic acid (30:67:3), methanol-36% acetic acid (95:5), methanol-formic acid (95:5) and water under reflux, sonication, Soxhlet extraction and ultrasonic agitation, immersion extraction, decoction, followed by TLC, CE, and HPLC analysis.

**Table 2:** The concentration variation of ferulic acid (**1**) in CX analyzed by different methods.

No.	Extraction solvent	Extraction method	Analytical method	Content (mg/g)	Ref.
1	95% ethanol	Reflux	TLCS	0.9395	[67]
2	70% ethanol	Sonication	HPCE	0.82~1.19	[66]
3	Methanol	Sonication	HPLC: PE-Pack C18 (4.6 mm × 150 mm, 5 µm), 1% glacial acetic acid:methanol (58:42), 0.5 mL/min, 313 nm	0.146~0.778	[68]
4	70% ethanol	Ultrasonic agitation	CE	0.82~1.19	[42]
5	95% ethanol	Soxhlet extraction	HPLC: Waters C18 (10 μm × 3.9 mm × 250 mm), 10% acetic acid:methanol (65:35), 1 mL/min, 320 nm	1.234~1.368	[69]
6	Methanol-water-36% acetic acid (30:67:3)	Sonication	HPLC: ODS C18 (250 mm × 4.6 mm), methanol:water:36% acetic acid (30:67:3), 1 mL/min, 322 nm	0.653~1.327	[51]
7	Methanol-36% acetic acid(95:5)	Sonication	HPLC: Kromasil C18 (250 mm ° 4.6 mm, 5 μm), acetonitrile:methanol:1% acetic acid (15:15:70), 0.6 mL/min	0.327~0.723	[70]
8	SFE	-	HPLC: Phenomenex (250 mm ° 4.6 mm, 5μm), methanol:water:glacial acetic acid (30:70:0.2), 1 mL/min, 320 nm	0.8	[71]
9	Water	Reflux	HPLC: DiamonsilTM C18 (250 mm ° 4.6 mm, 5 μm), methanol:water:glacial acetic acid (30:68:2), 1.0 mL/min, 320 nm	1.87~2.17	[72]
10	Methanol	Sonication	RP-HPLC: Inertsil C18 (250 mm × 4.6 mm, 5 μm), methanol:water:glacial acetic acid (35:65:0.5), 1.0 mL/min, 321 nm	1.00~1.14	[73]
11	Methanol-formic acid(95:5)	Sonication	HPLC: Kromasil C18 (250 mm × 4.6 mm, 5 μm), 1% acetic acid:acetonitrile, 1 mL/min, 320 nm	0.107~2.374	[74]
12	40% ethanol	Water bath reflux	HPLC: Lichrosorb C18 (4.6 mm × 250 mm, 5 μm), 1% acetic acid:methanol (70:30), 1 mL/min, 320 nm	1.141	[75]
13	70% methanol	Reflux	(1) HPLC: Agilent TC-C 18 (150 mm × 4.6 mm, 5 μm), acetonitrile:0.085% phosphoric acid (17:83), 1.0 mL/min, 316 nm(2) UPLC: Acquity UPLC HSS T3 (100 mm × 2.1 mm, 1.8 μm), acetonitrile:0.085% phosphoric acid (15:85), 0.3 mL/min, 316 nm	1.211.24	[76]

#### 2.1.4. Biological Activities

Aromatic acids in the herbs are frequently used in TCM formula to stimulate blood circulation and to remove blood stasis by preventing platelet aggregation and antithrombus [78]. The pharmacological studies of organic acids mainly focus on the main constituents, including compounds **1**, **2** and **12**. Compound **1**, a characteristic aromatic acid in CX, has been clinically used to treat angina pectoris and hypertensive diseases in China [79]. Previous investigations indicated that it could significantly improve blood fluidity, inhibit platelet aggregation, decrease serum lipids, prevent thrombus formation, protect neuron like pheochromocytoma cells (PC12), and exhibit strong antioxidant activity [28,80,81,82,83,84]. It was also reported that CX had anti-inflammatory action [85], could prevent ethanol-induced liver injury [86], contributed to the defense against viral infections including AIDS [87], as well as suppress the production of interleukin-8 (IL-8) which was the main cause of the local accumulation of neutrophils, and modulate various inflammatory reactions [85]. Compound **2** (3,4-dihydroxycinnamic acid) possesses anti-oxidant properties as it scavenges a number of reactive species, including 1,1-diphenyl-2-picrylhydrazyl (DPPH) free radical [88], peroxyl [89] and hydroxyl radicals [90], as well as superoxide anion, peroxynitrite and mutagenic compounds such as nitrosamines [91]. It also could inhibit 5-lipoxygenase activity [92], and inhibit protein kinase C, protein kinase (PKA) and nuclear factor-κB (NF-κB) activation induced by ceramides in U937 cells [93]. Compound **12** could inhibit carcinogenesis in the colon, liver, and tongue, and was against oxidative stress *in vivo* [94,95,96]. It has been claimed to modulate the glucose-6-phosphatase involved in glucose metabolism [97] and to reduce the risk of cardiovascular disease by decreasing oxidation of low density lipoprotein (LDL)-cholesterol and total cholesterol [98]. More recently, it has been reported that it can inhibit preadipocyte population growth, which may provide a proposed mechanism of reducing obesity [99].

### 2.2. Phthalides

#### 2.2.1. Chemical Structures

Phthalides are one kind of active compounds in CX with a phthalide parent nucleus and are used as characteristic components for quality control. Chemical structures of the main phthalides in CX are shown in Figure 2a,b, containing monomeric phthalides (compounds **19**–**51**) and phthalide dimers (compounds **52**–**66**) [9,10,100,101,102,103,104,105,106,107,108,109]. Takashi *et al.* [100,101,102,103] isolated about 30 phthalides from this herb in the 1990s, several of which were reported for the first time. Nowadays, some new phthalide and dimeric phthalides are isolated from CX in succession, such as 4,7-dihydroxy-3-butylphthalide (**43**), ligusticoside A (**63**) (a novel phthalide derivative with a lactone ring), chuanxiongnolide A (**64**), chuanxiongnolide B (**65**), 4,5-dihydro-3,1'-dihydroxy-3-pentylphthalide, and 4-pentylcyclohex-3-ene-1a,2b-diol [20,104,105]. Additionally, some compounds were found from this herb for the first time, such as *n*-hexadecanoic acid, daucosterol, 3-methylphthalide, and 3-butylidene-4,5-dihydro-2(1,3*H*)-1-isobenzofuranone [50,106].

**Figure 2:**
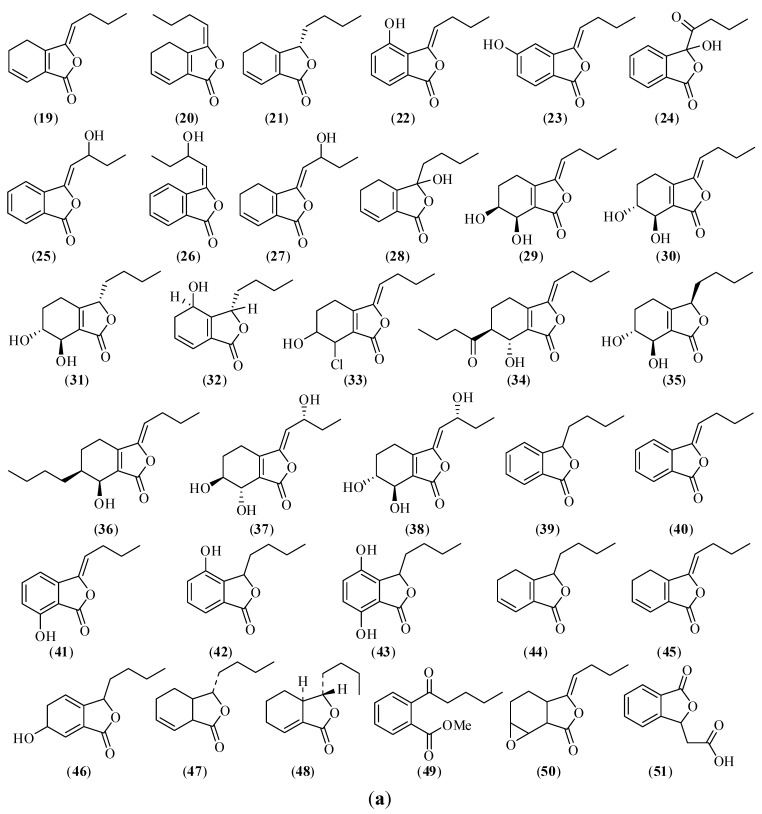
Chemical structures of main monomeric phthalides in CX: (**19**) *Z*-ligustilide; (**20**) *E*-ligustilide; (**21**) senkyunolide A; (**22**) senkyunolide B; (**23**) senkyunolide C; (**24**) senkyunolide D; (**25**) *Z*-senkyunolide E; (**26**) *E*-senkyunolide E; (**27**) senkyunolide F; (**28**) senkyunolide G; (**29**) senkyunolide H; (**30**) senkyunolide I; (**31**) senkyunolide J; (**32**) senkyunolide K; (**33**) senkyunolide L; (**34**) senkyunolide M; (**35**) senkyunolide N; (**36**) senkyunolide Q; (**37**) senkyunolide R; (**38**) senkyunolide S; (**39**) 3-butylphthalide; (**40**) 3-butylidenephthalide; (**41**) 3-butylidene-7-hydroxyphthalide; (**42**) 3-butyl-4-hydroxyphthalide; (**43**) 4,7-dihydroxy-3-butylphthalide; (**44**) 4,5-dihydro-3-butylphthalide; (**45**) 4,5-dihydro-3-butylidenephthalide; (**46**) 3-butylidene-6-hydroxy-5,6-dihydrophthalide; (**47**) cnidilide; (**48**) neocnidilide; (**49**) 2-(1-oxopentyl)-benzoic acid methyl ester; (**50**) *Z*-6,7-epoxyligustilide; (**51**) 3-carboxyethyl-phthalide.

**Figure 2 Cont.**
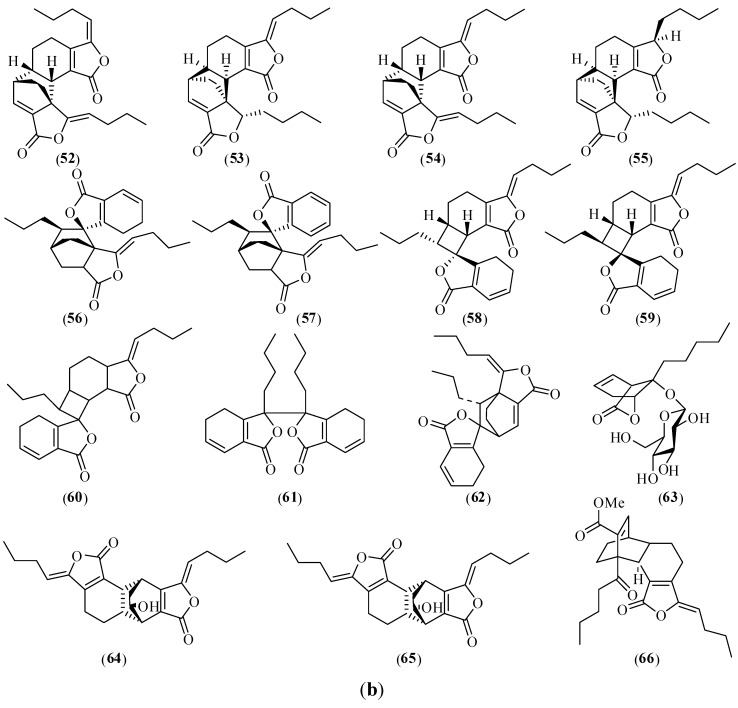
Chemical structures of main phthalide dimers in CX: (**52**) senkyunolide O; (**53**) senkyunolide P; (**54**) levistolide A; (**55**) 3,8-dihydrodiligustilide; (**56**) tokinolide B; (**57**) ansaspirolide; (**58**) riligustilide; (**59**) (*Z*,*Z'*)-6,8',7,3'-diligustilide; (**60**) angelicide; (**61**) *Z*,*Z'*-3,3',8,8'-diligustilide; (**62**) *Z*-ligustilide dimer E-232; (**63**) ligusticoside A; (**64**) chuanxiongnolide A; (**65**) chuanxiongnolide B; (**66**) wallichilide.

#### 2.2.2. Sample Preparation for Chemical Analysis

In most literatures the phthalide compounds in CX are extracted as a part of the volatile oil by steam distillation, solvent extraction, supercritical fluid extraction, circulation extraction coupling with adsorption and other methods [110]. Solid phase extraction could effectively enrich the phthalide components in CX, and eliminate the interference of other components [111]. In order to improve the extraction process and develop a greener extraction, a microwave-assisted extraction (MAE) method was investigated. The components of different polarity could be enriched in ionic liquids by choosing different extraction solvents. The ionic liquid MAE method takes a short time with high extraction efficiency and with less solvent and energy consumption [112].

A RP-preparative-HPLC method for the isolation of compound **30** from CX was described by Zhang *et al.* [113]. Because it is difficult for traditional methods including semi-preparative HPLC and CE to avoid the adsorption on the solid phase, they are not very suitable for the separation of compounds **19** and/or **20** and their analogues. High speed counter current chromatography (HSCCC) is a kind of liquid-liquid chromatography separation technique which is developed in recent years, and provides an advantage over the conventional column chromatography by eliminating the use of a solid support where an amount of stationary phase is limited and dangers of irreversible adsorption from the support are inevitably present. And now, it is accepted as an efficient preparative technique, and widely used for separation and purification of various natural and synthetic products [114]. Compounds **19** and **21**, the main components of volatile oil in CX, were separated in one step by HSCCC, and their purity could reach more than 95%, indicating that phthalides were fit to be separated and prepared by HSCCC with good resolution and high purity [115].

#### 2.2.3. Quantitative Analysis

Among the phthalides, compound **19** is frequently selected as a marker compound to evaluate the quality of CX. Concentration of ligustilide (**19** and/or **20**) in CX varies within the range of 5.672–15.700 mg/g in the reported literatures, which are quantified by a variety of methods (shown in Table 3) [116,117,118,119,120,121]. HPLC is the main analytical method for quantification of **19** and **20**. Although the content of **19** and/or **20** in CX was high, their instability and easy decomposition also brought about many problems for the determination. Compound **39** had a better stability than **19** and **20**. Shan *et al*. [122,123] determined the content of **39** in the volatile oil and crude herb of CX by HPLC (shown in Table 3). The results indicated the content of **39** in the volatile oil of CX was more than 17 times of that in CX crude herb.

HPLC with ultraviolet (UV) detection is increasingly used to analyze phthalides [124]. However, the sensitivity and selectivity of UV is insufficient for their direct identification in complex mixtures. GC-MS is a method that combines the features of gas-liquid chromatography and mass spectrometry, which was successfully used for simultaneous quantification of eight phthalides in essential oils from Si-Wu-Tang, Fo-Shou-San, Angelica Radix and CX [125]. However, the instability and structural similarity also cause difficulties in their analysis. Some of the volatile phthalides such as **19** are unstable and are easily changed into other phthalides through oxidation, isomerization, dimerization, or rapid decomposition at high temperature because of their active dihydrobenzene structure [126,127]. The dimeric phthalides, however, are also thermolabile; and retro-Diels-Alder reactions can easily take place even below 100 °C, which can’t be detected by GC-MS [128]. Therefore, application of HPLC coupled with MS is an attractive option to separate and identify such components [129,130,131].

#### 2.2.4. Biological Activities

Phthalides exhibit an equally broad spectrum of bioactivity, including modulation of the central nervous system and cardiac function, protection against brain ischemia, smooth muscle relaxation, inhibition of smooth muscle cell proliferation, anti-platelet aggregation, anti-angina activity, antibacterial, antifungal, antiviral and phytotoxic activity [132,133,134].

**Table 3:** The concentration variation of ligustilide (**19** and/or **20**) and butylphthalide (**39**) in CX analyzed by HPLC.

No.	Analytes	Extraction solvent	Extraction method	Analytical method	Stationary phase	Mobile phase	Flow rate (mL/min)	λ_max_(nm)	Content(mg/g)	Ref.
1	ligustilide	-	-	HPLC	Nova-Pak C18(3.9 mm ° 150 mm)	Methanol and water with 10% isopropanol (53:47)	0.8	280	15.7	[116]
2	ligustilide	Methanol	Sonication	HPLC	Luna 5 μm silica(150 mm ° 4.6 mm)	*n*-hexane:ethyl acetate:chloroform (92:3:5)	0.8	320	15.27 ± 1.86	[117]
3	ligustilide	Acetonitrile	Shaking up	RP-HPLC	Hypersil ODS2(4.6 mm ° 200 mm, 5 μm)	Methanol:acetonitrile:water (33:21:46)	0.8	275	347.9(in volatile oil)	[118]
4	ligustilide	Ethanol	Reflux	HPLC	C18(4.0 mm ° 200 mm, 5 μm)	Acetonitrile:water (both contain 0.1% acetic acid)	0.76	280	8.2	[119]
5	ligustilide	70% ethanol	Reflux	HPLC	ODS C18(4.6 mm ° 200 mm, 5 μm)	Methanol:water:acetic acid (75.0:24.8:0.2)	1.0	326	5.672~5.821	[120]
6	ligustilide	Ethanol	Sonication	HPLC	Alltima C18(4.6 mm × 150 mm, 5 μm)	Acetonitrile:water(60:40)	1.0	350	7.40	[121]
7	butylphthalide	Acetonitrile	Shaking up	RP-HPLC	Kromasil C18(250 mm × 4.6 mm, 5 μm)	sodium acetate (0.05 mol/L):acetonitrile(45:55)	1.0	228	131.2~138.3(in volatile oil)	[122]
8	butylphthalide	Ethyl ether	Sonication	RP-HPLC	Kromasil C18(250 mm × 4.6 mm, 5 μm)	Acetonitrile:acetic acid (pH 4.0, 45:55)	1.0	228	7.86~8.01	[123]

Among the phthalides, the main bioactive constituent focuses on compound **19**, which has been studied and demonstrated that it has properties of vasodilatation [135], antiasthmatic action [136,137], antiplatelet aggregation [138], analgesic [139], antithrombotic, antiproliferation [140], centrally acting on muscle relaxant [141], on central noradrenergic and/or γ-aminobutyric acid [GABA(A)] systems [142] In addition, recent studies showed that it exerted significant neuroprotective effects in transient forebrain ischemia, permanent cerebral focal ischemia, and chronic cerebral hypoperfusion [143,144,145]. Both constituents of **19** and **21** were reported to have an anti-inflammatory effect by inhibiting tumor necrosis factor-alpha (TNF-α) production and TNF-α-induced NF-κB activation *in vitro* [34,146], vasorelaxation activities in contractions to various contractile agents in rat isolated aorta [147]. Both compounds **19** and **40** can inhibit the abnormal proliferation of vascular smooth muscle cell (VSMC) induced by basic fibroblast growth factor (bFGF) [148], attenuate the suppressive effects of yohimbine (30 nmol, i.c.v.), methoxamine (200 nmol, i.c.v.) and a benzodiazepine inverse agonist FG7142 (10 mg/kg, i.p.) on pentobarbital sleep in group-housed mice [138].

### 2.3. Alkaloids

#### 2.3.1. Chemical Structures

**Figure 3:**
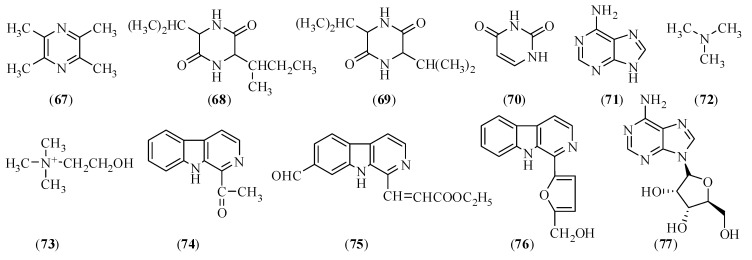
Chemical structures of main alkaloids in CX: (**67**) tetramethylpyrazine; (**68**) L-isobutyl-L-valine anhydride; (**69**) L-valine-L-valine anhydride; (**70**) uracil; (**71**) adenine; (**72**) trimethylamine; (**73**) choline; (**74**) 1-acetyl-β-carboline; (**75**) 1-β-ethyl acrylate-7-aldehydo-β-carboline; (**76**) pelolyrine; (**77**) adenosine.

Chemical structures of the main alkaloids from CX are shown in Figure 3 [45,63,149]. Tetramethylpyrazine (TMP, **67**), the molecular formula C_8_H_12_N_2_, is a characteristic alkaloid isolated from CX. Because compound **67** had the same pharmacological roles as CX and was first isolated from this herb, it was named chuanxiongzine by researchers.

#### 2.3.2. Sample Preparation for Chemical Analysis

Compound **67** in CX was extracted by SFE with a new class of low-pressure solvent (Phytosol), which is a non-chlorinated halothane solvent. During the extraction, just 30 mL of Phytosol solvent were needed, which could be recovered at room temperature [150]. CX total extract with high quality and stable yield could be obtained by another method of ethanol reflux and separation with macroporous resin. The extract yield was 0.6%, among which compound **67** accounted for 5%~7% [151]. Ultrasonic extraction was also used in the extraction of **67**, which is a new technology in extraction and separation of the active components in the herbal medicine in recent years. Compared with the conventional method of decoction, distillation and solvent immersion, the ultrasonic method had the advantages of a low extraction temperature, high biological activity of the product, less solvent consumption, time and energy savings [152]; extraction time of ultrafine vibration extraction technology (UVET) was 1/6, solvent amount was 4/7, the extraction rates of marker components (**1** and **67**) were 1.09 and 1.19 times, respectively [153].

#### 2.3.3. Quantitative Analysis

Compound **67** has also been considered as one of the main bioactive components and is used as a chemical marker for quality control of CX herb and CX-containing preparations (shown in Table 4) [8,154,155,156]. However, literature results suggest that the content of **67** was very low in CX, which was often less than the lower detection limit by HPLC [8]. Therefore, it should not be considered a suitable chemical marker for the quality control of CX herb and CX-derived herbal products.

**Table 4:** The concentration variation of TMP (**67**) in CX analyzed by different methods.

Extraction solvent	Extraction method	Analytical method	Content (mg/g)	Ref.
Benzene, ethyl ether, and ethyl acetate	Refluxing	HPLC-DAD	1.2 ° 10^−4^	[8]
Petroleum ether	Counter current	RP-HPLC	0.12 ° 10^−3^~0.87 ° 10^−3^	[154]
Ethanol	Sonication	HSCCC	0.042	[155]
80% ethanol (containing 5% acetic acid)	Sonication	HPLC	0.01256~0.07252	[156]

Total quaternary ammonium alkaloids in CX were determined by neutralization titration, acidic dye colorimetry, and Reinecke salt colorimetry. The results from acidic dye colorimetry showed that among three kinds of CX species (Nai-CX, Shan-CX, and CX), the content of total alkaloids in CX was the highest [157]. Acidic dye colorimetry, however, had weak sensitivity and complex sample processing, which could easily cause error. Therefore, the content of total alkaloids in CX was determined by Reinecke salt colorimetry. Determined by this method, the content of total alkaloids in CX was 0.265%. However, it was 0.237% determined by acidic dye colorimetry [158]. In order to inherit and develop our traditional “*Paozhi*” technology and better serve for clinic, the content of alkaloid in CX and CX-processed products (Cu-CX and Jiu-CX) were determined by neutralization titration and TLC scanning method [159]. The content of compound **67** in Jiu-CX was lower than that in CX raw herb, but the content of total alkaloids was higher. It was probably because the melting point of **67** was 80~82 °C, so it was easily sublimed when it was heated. The contents of total alkaloids and compound **67** in Cu-CX both were higher than that in CX raw herb, which might be attributed to the salt formation from alkaloid and acid which could be easily extracted.

#### 2.3.4. Biological Activities

Compound **67**, an active compound of CX, has been reported to be an agent against platelet aggregation [160], a vasodilator with calcium channel blocking activity [161], a scavenger of superoxide anion in human polymorphonuclear leukocytes [162], and a chemical with anti-portal hypertensive and hepatoprotective effects [163,164]. Recently, it was discovered to have protective effects on multiple organs and systems [165], to alleviate kidney and brain damage induced by ischemia/perfusion in rats via scavenging oxygen-free radicals [166], to postpone chronic renal allograft dysfunction associated with cold ischemia injury and chronic allograft rejection but to have no evident hepatic side effects [167]. Moreover, compound **67** could significantly suppress oxidative stress and attenuate cell death in neuronal cultures induced by glutamate analogue and iron-mediated oxidative stress [168,169]. It also shows anti-apoptotic effect in rabbit ischemic spinal cord and hydrogen peroxide (H_2_O_2_)-induced PC12 [170,171].

### 2.4. Polysaccharides

#### 2.4.1. Chemical Structures

The components in CX have drawn the attention of researchers since around the 1950s. However, researchers have only started the study of polysaccharides in CX in 2004. Since then, studies have been gradually expanded. Four homogeneous polysaccharides were obtained from the water extract of CX for the first time, with the molecular weights of 3.1 × 10^4^, 5.2 × 10^4^, 9.0 × 10^4^, and 3.6 × 10^4^, respectively [172,173]. Polycose in CX is composed by arabinose (Ara), galactose (Gal) and glucose (Glc), in a molar ratio of 1:1.4:7.9, determined by filter paper chromatography, TLC, and vapor phase chromatography [174]. Additionally, three purified polysaccharides (LCA, LCB, and LCC) were obtained [38]. The estimated weight is around 2.83 ° 10^4^ Da, 1.23 ° 10^4^ Da, and 6.31 ° 10^4^ Da, respectively. LCA is α-Glc linkaged pyranose, composed by Ara, Gal, and mannose (Man). LCB consists of Ara, Glc, Gal, and Man; while LCC comprises Ara, Glc, and Gal. The result of Sun *et al.* [175] were in agreement with other literatures, except for the LCP-3, which still included rhamnose, galacturonic acid except Glc, Gal, Ara and Man, as determined by HPLC method after derivatization.

#### 2.4.2. Sample Preparation for Chemical Analysis

At present, there are many methods for the extraction of polysaccharides, including water diffusion, osmosis, reflux, basic, ultrasonic, microwave, enzyme, supercritical fluid extraction (SFE) and macroporous resin methods (shown in Table 5) [176,177,178,179,180,181,182]. As indicated, both the pectinase method and cellulose method had higher extraction rates of polysaccharide from CX. Additionally, the enzyme method had mild reaction conditions and relatively simple operation compared with other methods.

**Table 5:** The extraction methods of polysaccharide in CX.

Extraction method	Optimum technology	Extraction rate (%)	Ref.
Ultrasonic	Ultrasonic time: 40 min; ultrasonic power: 400 W; solid to liquid ratio: 1:10; extraction times: 2	2.74	[176]
Pectinase	Compound pectinase: 1%; temperature: 60 °C; pH value: 3.5; the heating time: 150 min	11.3	[177]
Basic	Extraction temperature: 95 °C; Extraction time: 150 min; the concentration of NaOH: 0.8 mol/L; solid to liquid ratio: 1:200 g/mL	2.69	[178]
Enzymic	Cellulase: 0.15%; the compound pectinase: 10%; time: 210 min; pH: 3.4; temperature: 60 °C	3.03	[179]
Microwave assisted	Microwave power: 231 W; solid to liquid ratio: 1:40; extraction time: 10 min	3.06	[180]
Cellulose enzymic	Cellulase: 0.25%; time: 120 min; pH value: 4.0; temperature: 50 °C.	7.26	[181]
Basic	Extraction temperature: 90 °C; extraction time: 4 h.	6.7	[182]

During the extraction of polysaccharide in CX, the color of the extract was very deep, which might interfere with the quantitative determination. Therefore, it is necessary to decolorize the extract using absorbite, H_2_O_2_ and macroporous adsorptive resins. The results showed that macroporous adsorptive resins S-8 had the best decolorization effect. The rate of decolorization was 92.7%, and the retention rate of polysaccharide of CX was 93.0% [183].

#### 2.4.3. Quantitative Analysis

Polysaccharides generally should be hydrolyzed before quantitative determination. Then, reducing sugars can be determined by the carbolic acid method, sulphuric acid method, anthrone method, salicylic acid colorimetric method, Fehling reagent method or phenol method. Through weight calculations, the yield of crude polycose was 6.7%. The polycose was 4.11% as determined by the carbolic acid method, sulphuric acid method, and anthrone method [174]. However, manual operation, which can’t accomplish rapid monitoring and on-line analysis, is slow and involves more consumption of reagent. Therefore, a simple and rapid method of flow injection on-line hydrolysis spectrophotometry for the determination of polysaccharide in CX was developed [184]. It has been applied to a rapid determination of polysaccharide in CX with satisfactory results. The content of polysaccharide in CX was 2.28%.

#### 2.4.4. Biological Activities

It was shown that all purified polysaccharides (LCA, LCB, and LCC) exhibited antioxidation and cytotoxicity. Among them, LCB has the highest antioxidant and cytotoxic activity, and scavenging ability on hydroxyl radicals. It is possible that LCB can be explored as a novel potential antioxidant and cytotoxic natural bioactive macromolecule [38]. Additionally, the polysaccharides of CX have antibacterial activities and promote cell growth [185].

### 2.5. Ceramides and Cerebrosides

#### 2.5.1. Chemical Structures

**Figure 4:**
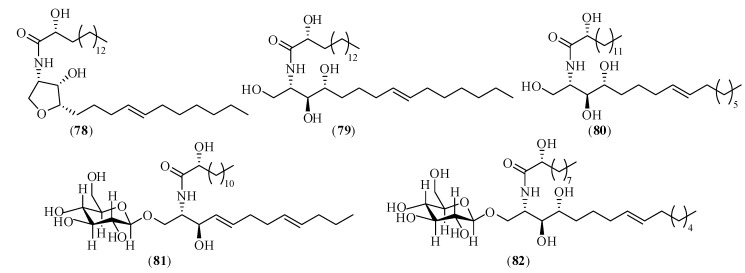
Chemical structures of the main ceramides and cerebrosides in CX: (**78**) (2*R*)-2-hydroxy-*N*-[(2*S*,3*S*,4*R*,8*E*)-1,3,4-trihydroxypentadec-8-en-2-yl]heptacosanamide; (**79**) (2*R*)-2-hydroxy-*N*-{(3*S*,4*S*,5*S*)-4-hydroxy-5-[(4*E*)-undec-4-en-1-yl]tetrahydrofuran-3-yl}heptacosanamide; (**80**) (2*R*)-2-hydroxy-*N*-[(2*S*,3*S*,4*R*,8*E*)-1,3,4-trihydroxyicos-8-en-2-yl]tetracosanamide; (**81**) (2*R*)-*N*-[(2*S*,3*R*,4*E*,8*E*)-1-(β-D-glucopyranosyloxy)-3-hydroxy-dodeca-4,8-dien-2-yl]-2-hydroxydocosanamide; (**82**) (2*R*)-*N*-[(2*R*,3*S*,4*R*,8*E*)-1-(β-D-gluco-pyranosyloxy)-3,4-dihydroxyoctadec-8-en-2-yl]-2-hydroxyhexadecanamide.

Ceramides and cerebrosides, two families of sphingolipids, are important components of a wide variety of tissues and organs in biological systems (fungi, plants, animals, and marine organisms). Chemically, ceramide usually consists of a long-chain sphingosine or sphingol and an amide-linked long-chain fatty acid; cerebrosides are composed of a hexose and a ceramide moiety. Yang *et al.* [186] isolated three ceramides (compounds **78**–**80**) and two cerebrosides (compounds **81**, **82**) from the petroleum ether extract of CX in 2009. Amongst them, compounds **78** and **79** were new ceramides, compound **80** was a known ceramide, **81** and **82** were two known ceramides, but **80**–**82** were isolated from CX for the first time. Their structures are shown in Figure 4.

#### 2.5.2. Biological Activities

The content of ceramides and cerebrosides in plants is very low. However, they have strong biological activities. A growing amount of evidence has indicated that ceramides and cerebrosides have a wide range of biological functions regulating cell growth and variation, participating in protein secretion and immunologic processes, protecting nerves, angiocarpy and liver cells. Biologically, cerebrosides have been proven to serve as structural supports and texture determinants of cell membranes, and to act as mediators of biological events [186,187,188].

### 2.6. Other Compounds

**Figure 5:**
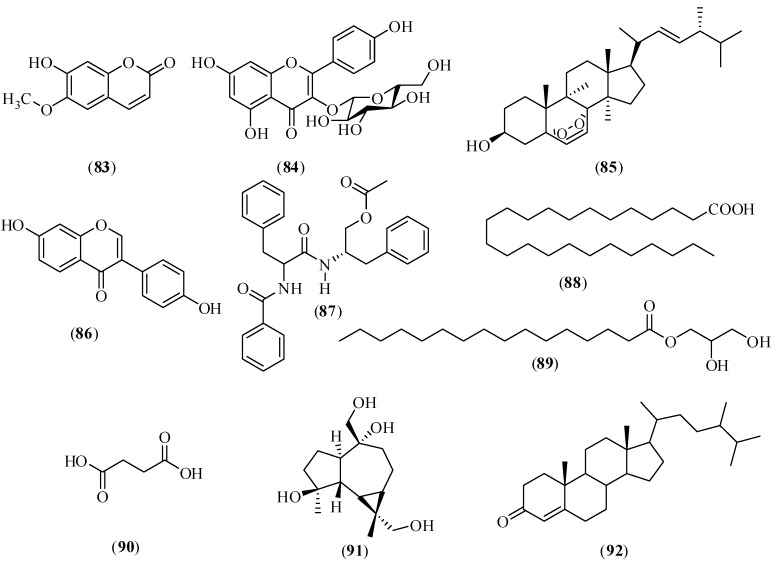
Chemical structures of other compounds in CX: (**83**) scopoletin; (**84**) astragalin; (**85**) ergosterol peroxide; (**86**) daidzein; (**87**) aurantiamide acetate; (**88**) lignoceric acid; (**89**) monopalmitin; (**90**) succinic acid; (**91**) (−)-alloaromadendrane-4β,10α,13,15-tetrol; (**92**) campest-4-en-3-one.

In addition to the above five types of components, CX also contains other compounds. Chemical components of CX aerial parts were studied using various separation methods, such as silica gel column chromatography, ODS column chromatography, and Sephadex LH-20. Twenty five compounds were separated and purified from a 80% ethanol extract of CX aerial parts. According to the physical and chemical properties, six compounds which were isolated from CX for the first time were identified using MS, ^1^H-NMR, ^13^C-NMR, distortionless enhancement by polarization transfer (DEPT), heteronuclear single quantum coherence (HSQC) and heteronuclear multiple-bond correlation (HMBC). Scopoletin (**83**) and astragalin (**84**) were separated from the ethyl acetate fraction of the dried 80% ethanol extract of CX aerial parts; ergosterol peroxide (**85**), daidzein (**86**), aurantiamide acetate (**87**) and lignoceric acid (**88**) were separated from the petroleum ether fraction [189]. Eleven compounds were separated and purified from 95% ethanol extract of CX by Hao *et al*. by repeated purification on a silica gel column, Sephadex LH-20 column chromatography, preparative TLC, and semi-preparative HPLC. Monopalmitin (**89**) and succinic acid (**90**) were separated from the petroleum ether and chloroform fractions of the dried 95% ethanol extract of CX [190]. Furthermore, the dried methanol extract of the 80% ethanol crude extract was subjected to column chromatography on silica gel eluted with a gradient system of chloroform/methanol (100:0 to 80:20) to afford 12 fractions. (−)-Alloaromadendrane-4β,10α,13,15-tetrol (**91**) was separated and purified from the fraction eluted from silica gel with chloroform/methanol (20:1), followed by reverse phase column chromatography eluted with methanol/H_2_O (30:70). The fraction eluted with petroleum ether/ethyl acetate (15:1 to 12:1), was further subjected to column chromatography on silica gel to yield campest-4-en-3-one (**92**) [191]. Isolated compounds **91** and **92** were tested for their abilities to inhibit the growth of microorganisms, and the results showed that they all had mild antimicrobial activities. Their structures are shown in Figure 5.

## 3. Analytical Methods for Simultaneous Determination of Different Types of Chemical Compounds

### 3.1. HPLC and HPLC-MS

According to the development profile of CX (Table 1), fresh CX samples (October 8, 2002; October 30, 2002; December 2, 2002; January 2, 2003; February 11, 2003; March 9, 2003; April 11, 2003; April 17, 2003; April 24, 2003; May 2, 2003; May 9, 2003; May 15, 2003; and May 20, 2003) were collected and analyzed using HPLC-UV, and the results showed that all samples contained compounds **13**, **19** and **21** as the major and compounds **1** and **40** as the minor components, while only trace amounts of components **54** and **58** were found in some of the samples. Compounds **29** and **30** were not detected in fresh CX herb, but detected in commercial dried and/or processed CX, which suggests they might be generated by the chemical conversion of the major phthalide **19** during processing and/or storage. Both individual and total contents of all main bioactive components gradually increased from the beginning of October to the middle of next April; and the weight of a single rhizome reached a plateau at the end of May, whilst the content of the major ingredients (**13**, **19** and **21**) peaked in the middle of April. Therefore, the optimal harvest time for this herb is in the period from the middle of April to the end of May when the node of the plant stem becomes swollen and purplish [16].

To date, HPLC has been extensively applied to the qualitative and quantitative analysis of chemical constituents of CX, including organic acids, phthalides, and alkaloids, and remains the prime method in compositional analysis of this herb. Representative HPLC methods developed for the simultaneous determination of chemical analysis of CX are summarized in Table 6 [8,59,110,119,192,193,194,195,196,197,198,199,200,201]. From it we can see that most HPLC separations of CX were carried out on reversed phase C18 columns, and some were on reversed phase C8 columns. In recent years, hyphenated HPLC techniques like HPLC-MS coupled with DAD, UV, and ESI focusing on structure elucidation have become widely available. With the aid of these modern spectroscopic techniques, multiple components of CX were simultaneously determined and identified. Phthalide dimers, present in smaller amounts, also have been identified by HPLC-UV and HPLC-MS. Stereochemical features of some phthalide monomers have been determined by detailed spectroscopic studies for the first time [202]. Additionally, some phthalides of CX are unstable and difficult to analyze by GC-MS. But they can unambiguously be identified by optimized LC-MS, and characteristic fragments of them can be obtained using homemade reference standards [203]. What is more, as a typical form of multi-dimensional separation system, a comprehensive two-dimensional LC system has been widely used to characterize and separate biomolecules, polymers, and other complex mixtures due to its high peak capacity, powerful separation and resolution ability since it appeared in 1978 [204]. The comprehensive two-dimensional system can provide maximum information with minimal amounts of material and allow rigorous quantitative interpretation of the results. Therefore, a comprehensive two-dimensional LC separation system based on the combination of a CN column and an ODS column was developed for the separation of components of CX [205]. Two columns are coupled by a two-position, eight-port valve equipped with two storage loops and controlled by a computer. More than 52 components in the methanol extract of CX were resolved and 11 (compounds **1**, **2**, **3**, **6**, **19**, **29**, **30**, **39**, **40**, **42**, and **44**) of them were preliminary identified according to their UV and mass spectra.

**Table 6:** HPLC methods developed for simultaneous chemical analysis of CX.

No.	Analytes	Detection mode	Stationary phase	Mobile phase	Ref.
1	**1**, **6**, **29**, **30** and **67**	HPLC-MS	Zorbax SB-C18(250 mm × 4.5 mm, 5 µm)	Methanol:water:acetic acid (45:55:0.5, v/v/v)	[8]
2	**1**, **6**, **13**, **19**, **21**, **27**, **29**, **30**, **31**, **39**, **40**, **47**, **48**, **53**, **54**, **56** and **58**	HPLC-DAD-MS	Waters symmetry C18(150 mm × 2.1 mm, 5 µm)	0.25% aqueous acetic acid and methanol	[192]
3	**1** and **19**	HPLC-MS	C_18_(4.0 mm × 200 mm, 5 µm)	Acetonitrile with 0.1% acetic acid and 0.1% acetic acid	[119]
4	**1**, **2**, **3**, **8** and **12**	HPLC-UV	Zorbax SB-C18(250 mm × 4.6 mm, 5 µm)	Water with 0.1% acetic acid and methanol	[59]
5	**19**, **21**, **29** and **30**	HPLC-DAD	Eclipse XDB-C_8_(4.6 mm i.d. × 150 mm)	Methanol and water with 1% formic acid	[110]
6	**1**, **6**, **13**, **19**, **21**, **29**, **30**, **40**, **48**, **54**, **58** and **67**	HPLC-UV	Waters symmetry C18(150 × 4.6 mm, 5 µm)	0.25% aqueous acetic acid and methanol	[193]
7	**1**, **13**, **19**, **20**, **21**, **27**, **29**, **30**, **31**, **39**, *E*-**40**, *Z*-**40**, **47**, **53**, **54**, **56**, **58**, **60**, **61** and **62**	HPLC-DAD-MS	Alltima C18(4.6 mm × 250 mm, 5 µm)	0.5% acetic acid in water and acetonitrile	[194]
8	**21**, **19**, **48**, **39**, **62**	HPLC-MS^n^	Eclipse XDB-C_18_(4.6 mm × 150 mm, 5 µm)	0.25% acetic acid and methanol (containing 0.25% acetic acid)	[195,196]
9	**1**, **13**, **19**, **20**, **21**, **27**, **29**, **30**, **39**, *E*-**40** and *Z*-**40**	HPLC-ESI-MS	Alltima C18(4.6 mm × 250 mm, 5 µm)	Water and acetonitrile	[197]
10	**1**, **42** and 6,7-di-hydroxyligustilide	HPLC-DAD	Shinwa-ODS(250 mm × 4.6 mm, 5 µm)	Methanol and 0.1% acetic acid	[198]
11	**1**, **19**, **39**, **40** and **67**	RP-HPLC-DAD	Grace Smart RP C_18_(250 mm × 4.6 mm, 5 µm)	Acetonitrile and 0.1% phosphoric acid	[119]
12	**19** and **21**	HPLC-DAD	Zorbax SB-C_18_(4.6 mm × 250 mm, 5 µm)	Acetonitrile and 1% acetic acid	[200]
13	**1**, **2**, **12**, **19**, **29**, **30** and **40**	HPLC-DAD	Alltima-C_18_(250 mm × 4.6 mm, 5 μm)	0.2% aqueous formic acid and acetonitrile	[201]

### 3.2. GC-MS

Nowadays, modern sample preparation techniques in analytical chemistry are characterized by simplification, miniaturization, high enrichment and minimization of sample and solvent amounts. The volatile compounds in CX are recognized as an important part for its pharmacological activities [14]. For the analysis of the volatile compounds, some publications are available using GC-MS following steam distillation extraction, SFE, and other extraction methods [206]. Representative GC-MS methods developed for the chemical analysis of CX are summarized in Table 7 [20,59,108,207,208,209,210,211,212,213]. According to different chromatographic conditions, several or even hundreds of components of CX can be simultaneously determined by this method. The column temperature commonly uses programmed temperatures.

Among these methods, GC-MS sometimes was used to analyze and identify the volatile chemical components of CX by the combination of chemometric local resolution techniques such as subwindow factor analysis (SFA), orthogonal projection resolution (OPR) [209], heuristic evolving latent projections (HELP), the overall volume integration method [212], and other methods. With the help of chemometric approaches, the purity of chromatographic peaks can be identified. The combination of GC-MS with chemometric local resolution methods could greatly improve the chromatographic separation ability by means of mathematical approaches, indicating the reliability and practicability of these combined techniques. A headspace solid-phase microextraction (HS-SPME) method followed by GC-MS was described and a comparison between HS-SPME-GC-MS and steam distillation (SD)-GC-MS methods was made [213]. The results showed that HS-SPME method could achieve comparable results (73 compounds) with those by SD method (about 50 compounds), using much less sample, shorter extraction time and a simpler procedure. Additionally, effective components **19**/**20** and **40** were screened and identified using a cell membrane chromatography (CMC) and a simple capillary GC-MS method [14,206]. The volatile oils of 23 CX samples from four different regions were analyzed by comprehensive two-dimensional GC/time-of-flight/MS (GC-TOF-MS) [214]. The group-type separation of four terpenoids (monoterpenes, oxygenated monoterpenes, sesquiterpenes, and oxygenated sesquiterpenes) and phthalides was well accomplished based on a DB-Petro × DB-17 column system. With the MS library search, 215 compounds were tentatively identified based on the NIST database and 43 compounds of them were confirmed. Twenty three samples were apparently classified into four groups by partial least square-discriminant analysis (PLS-DA). All the results indicated that phthalides exerted a great influence on the chemical and biochemical classifications of CX.

**Table 7:** GC-MS methods developed for the chemical analysis of CX.

No.	Analytes	Detection mode	Stationary phase	Temperature	Ref.
1	**19**, **39**, **40**, **47**, **48** and senkyunolide	HP 5890 SERIES I GC	Gross-Linked Methyl Silicone Gum Phase (25 m × 0.2 mm)	Column: 80 °C; injector and detector: 250 °C; source: 200 °C; interface: 280 °C	[207]
2	**19**, **20**, **21**, **22**, **27**, **29**, **30**, **39**, **40** and **48**	HP6890 (GC) and a mass selective detector (HP5973)	HP-5 MS capillary column(30 m × 0.25 mm, 0.25 µm)	Column: 80 °C–280 °C; injector: 250 °C; source: 250 °C	[59]
3	45 components were identified.	HP5988A GC-MS	SE-30 capillary column(30 m × 0.25 mm, 0. 25 µm)	Column: 90 °C–250 °C; injector: 260 °C	[208]
4	About 127 chemical components be separated and 81 of them identified.	ShimadzuGC-17A	OV-17 capillary column(30 m × 0.25 mm)	Column: 40 °C–230 °C; injector: 250 °C; source: 230 °C	[209]
5	59 components were identified.	Agilent 6890N 5973N GC-MS	HP-1 capillary column(30 m × 0.25 mm)	Column: 40 °C–230 °C; injector: 280 °C; source: 230 °C; interface: 280 °C	[210]
6	**19** and **21**	Shimadzu GC-14B	SE-54 quartz capillary column(50 m × 0.2 mm)	Column: 240 °C; injector and detector: 280 °C	[211]
7	52 volatile chemical components were determined.	Agilent 6890N 5973N GC-MS	HP-5MS capillary column(30 m × 0.25 mm)	Column: 60 °C–250 °C; injector: 250 °C; source: 230 °C; interface: 280 °C	[212]
8	73 compounds were identified.	HP 5973 GC-MSD	HP-INNOWAX(30 m × 0.25 mm, 0.25 µm)	Column: 50 °C–210 °C; injector: 250 °C; source: 250 °C; interface: 280 °C	[213]
9	62 components were identified.	Trace MS 2000 GC-MS	DB-5 capillary column(0.25 mm × 30 m, 0.25 µm)	Column: 50 °C–240 °C; injector: 270 °C; source: 200 °C; interface: 250 °C	[20]
10	52 compounds were identified.	HP 6890 N GC	HP-5(30 m × 0.32 mm, 0.25 µm)	Column: 40 °C–100 °C; injector: 260 °C; source: 200 °C; interface: 220 °C	[108]

### 3.3. CE

CE is increasingly recognized as an important analytical separation technique because of its speed, efficiency, reproducibility, ultra-small sample volume, less consumption of solvent and ease of clearing up the contaminants [215]. CZE, which was first reported in 1981 [216], is an important model of CE. The content of compound **1** in CX was easily determined within 15 min with no pretreatment and interference by CZE [42]. Because of the complex constituents of CX, the column is easily polluted by the volatile oil, saccharide, and other components of this herb when detected by HPLC, thus leading to shortened life expectancy. However, those shortcomings can be overcome by HPCE. In recent years, HPCE has been developed as a powerful multi-dimensional separation technique [217], which is a hybrid technique that combines the advantages of HPLC and CE, also one of the very suitable analysis techniques for the multi-components analysis of TCM. This method has been recorded by the appendix of Chinese Pharmacopoeia (the 2005 edition), indicating it has become an official analysis technique for TCMs. The chemical composition spectrum of aqueous solution of CX was analyzed by HPCE, and compound **67** was identified [218]. However, in practice, when capillary electrochromatography (CEC) is used without pressure, often on a commercial CE instrument, there are problems associated with bubble formation in CEC, occurring initially in the unpacked section of the capillary, probably as a result of differences in velocity of the liquid eluent between the packed and unpacked sections of the capillary and column dry out [219]. The use of supplementary pressure has proved effective to stabilize the flow conditions. Compared with traditional HPLC and CE, the mobile phase in the pressurized CE (pCEC) system is driven by a pressurized flow and an electroosmotic flow simultaneously, reducing band broadening and improving separation efficiency. Now pCEC has become an attractive technique for pharmaceutical analysis because of its combination of the inherent advantages of two major separation techniques [220,221]. In addition, with amperometric detection (AD), CE-AD affords high sensitivity and good selectivity for electroactive species. Bioactive ingredients of CX including compounds **1**, **2**, **3**, **4**, **5**, **6**, **8**, and **12** were determined by CE-AD as marker compounds, and the characteristic “electrochemical profiles” of CX was studied [60,215].

## 4. Fingerprinting

Fingerprint analysis is considered as one of the most powerful approaches in quality control of TCMs. Conventional research focuses mainly on the determination of the most active components, while fingerprinting can offer characterization of a complex system with a degree of quantitative reliability, which is consistent with the theory that all the components, not just the few active compounds. in TCMs are held to be responsible for the beneficial effects Furthermore, fingerprint analysis can be used for identifying and assessing the authenticity, stability, plant anatomy, geographical origins, and harvest time of medicinal herbs. Nowadays, chromatographic techniques have been widely used in fingerprint analysis for quality control of CX, such as HPLC [222,223], HPCE [218,224], pCEC [221], hydrophilic interaction chromatography (HILIC)-RPLC [30], ^1^H-NMR-HPLC [225], HPLC-DAD-MS [226], and GC-MS [227,228].

Fingerprint similarity analysis of CX from three producing areas (Jiangxi, Sichuan, and Guangdong province of China) was studied by HPLC, and the peak areas of fifteen common components in their chromatograms were used to construct the fingerprints [222]. Principal component analysis (PCA) was applied to the fingerprint. It showed that the original region will cause significant differences. A vector of differences was defined between two fingerprints. The scalar mean of the different vector was taken as a statistic and both the *t*-test and Bayesian hypothesis testing were implemented to provide a one-to-one comparison of the fingerprints. Compared with PCA, correlation coefficient and vector cosine, the new method offers a better differentiation of the similarity or difference between the fingerprints from same sample of CX. Xie *et al*. [221] successfully developed a characteristic fingerprint by using pCEC and HPLC simultaneously for identifying raw CX herb. Two mathematical methods, correlation coefficient and the included angle cosine were applied for quantitative studies of the similarity of 10 batches of CX. Characteristics of pCEC and HPLC methods used to develop TCM fingerprint were summarized. It was proved that pCEC could be used as an alternative or supplementary technique for the development of fingerprint analysis of TCM through HPLC procedures. A binary chromatographic fingerprint analysis using HILIC and RPLC was developed to gain more chemical information about polar compounds and weakly polar compounds, which was used to construct a chromatographic fingerprint of CX [30]. Data from the analysis of CX samples were processed with similarity analysis, with correlation coefficients and congruence coefficients. To compare the quality of 14 samples, the change trend of similarity among the 14 samples calculated with four different methods (correlation coefficients and congruence coefficients with median and average data) was described. The production area of CX was relatively localized, mainly in Sichuan Province, and there was no obvious difference in the quality of samples from several areas in Sichuan Province, e.g., samples from Peng region and Guan region showed quality consistent with those from a genuine production area (Dujiangyan region). The technique of ^1^H-NMR and HPLC fingerprinting analysis is rapid, reproducible and stable with time for the authentication of medicinal plant species [229,230]. The ^1^H-NMR fingerprints of fractionated non-polar extracts (control substance for a plant drug, CSPD A) from CX of seven specimens from different sources were measured on Fourier Transform-NMR spectrometer and assigned by comparing them with the ^1^H-NMR spectra of the isolated pure compounds. The ^1^H-NMR fingerprints showed exclusively characteristic resonance signals of the major special constituents of the plant. Although the differences in the relative intensity of the ^1^H-NMR signals due to a discrepancy in the ratio of the major constituents among these samples could be confirmed by HPLC analysis, the general features of the ^1^H-NMR fingerprint established for an authentic sample of the rhizomes of CX exhibited exclusive data from those special compounds and could be used for authenticating CX species [225].

In the study of TCM fingerprints, the combination of chromatography and MS has better specificity than a single chromatography, and provides a more reliable basis for quality control. All the fingerprint results showed that place of origin significantly influenced the kinds and content of components in crude TCMs, and hence affected their quality.

## 5. Conclusions

We have summarized the recent progress in the chemical analysis of CX. Organic acids, phthalides, alkaloids, polysaccharides, ceramides and cerebrosides are believed to be the main bioactive constituents of CX. A variety of technologies have been used for the qualitative and quantitative analyses of these compounds in CX and its preparations. Currently, HPLC, HPLC-MS and GC-MS are mostly used for CX analysis. However, the qualitative information using HPLC-MS under multiple reaction monitoring (MRM) mode is not enough to elucidate the structure. Though in the full-scan mode this information can be obtained, the lack of sensitivity and the lack of compound databases and mass-spectral libraries may be obstructive to the qualitative analysis. So far, no satisfactory method has been reported for the simultaneous analysis of all the major components of CX. A novel approach to chromatographic separation is ultra performance liquid chromatography (UPLC), which is based on the use of columns with smaller packing and operated at higher pressures [231]. Compared with traditional HPLC, UPLC provides a higher peak capacity, greater resolution, increased sensitivity and higher speed of analysis. When coupled with orthogonal quadrupole time-of-flight mass spectrometry, UPLC-Q-TOF-MS provides several advantages in the separation of complicated samples such as the field of TCMs, metabolomics, and drug metabolism [232,233]. The advantages of its higher resolution and the accuracy in mass measurements make it a powerful tool for identification of the analytes, therefore, UPLC-Q-TOF-MS facilitates the rapid and sensitive characterization of CX extracts even when pure standards are not available. Furthermore, MRM using tandem quadrupole mass spectrometry (TQ-MS), which monitors both the specific precursor ions and product ions of each metabolite, is a standard technique in targeted metabolomics, as it enables high sensitivity, reproducibility and a broad dynamic range. By UPLC-TQ-MS analysis, more analytes and metabolites can be quantified in each crude herb extract or its biological sample [234,235]. Given these advantages, we can expect that they will find applications in many aspects of CX analyses, such as chemical fingerprinting, which globally addresses the organic acids, phthalides, alkaloids and other compounds; the simultaneous determination of most major organic acids, phthalides and alkaloids for the quality control. All these studies are critically important for the quality control of CX, so as to ensure its safety and efficacy in clinical applications. What is more, the characteristics of UPLC-Q-TOF-MS, UPLC-TQ-MS, and other techniques such as UPLC-high definition mass spectrometry (HDMS) will be the method of choice for the *in vivo* metabolism, metabolomics and pharmacokinetic studies of CX.
